# Naïve Bayes Classifiers and accompanying dataset for *Pseudomonas syringae* isolate characterization

**DOI:** 10.1038/s41597-024-03003-x

**Published:** 2024-02-07

**Authors:** Chad Fautt, Estelle Couradeau, Kevin L. Hockett

**Affiliations:** 1 Department of Plant Pathology and Environmental Microbiology, Pennsylvania State University, University Park, Pennsylvania USA; 2 Department of Ecosystem Science and Management, Pennsylvania State University, University Park, Pennsylvania USA; 3 Intercollege Graduate Degree Program in Ecology, Pennsylvania State University, University Park, Pennsylvania USA

**Keywords:** Pathogens, Infectious-disease diagnostics, Environmental microbiology

## Abstract

The *Pseudomonas syringae* species complex (PSSC) is a diverse group of plant pathogens with a collective host range encompassing almost every food crop grown today. As a threat to global food security, rapid detection and characterization of epidemic and emerging pathogenic lineages is essential. However, phylogenetic identification is often complicated by an unclarified and ever-changing taxonomy, making practical use of available databases and the proper training of classifiers difficult. As such, while amplicon sequencing is a common method for routine identification of PSSC isolates, there is no efficient method for accurate classification based on this data. Here we present a suite of five Naïve bayes classifiers for PCR primer sets widely used for PSSC identification, trained on in-silico amplicon data from 2,161 published PSSC genomes using the life identification number (LIN) hierarchical clustering algorithm in place of traditional Linnaean taxonomy. Additionally, we include a dataset for translating classification results back into traditional taxonomic nomenclature (i.e. species, phylogroup, pathovar), and for predicting virulence factor repertoires.

## Background & Summary

The *Pseudomonas syringae* species complex (PSSC) has been co-evolving with plants since before the emergence of angiosperms^1^, and has diversified into one of the most economically important groups of plant pathogens in the world, with a collective host range spanning almost every major food crop grown today^2^. Critically, while there are many pathogens within PSSC, there is also a wide range of virulence exhibited throughout the species complex, including non-pathogenic plant epiphytes and strains isolated from rain and snowpack with no known pathogenicity to plants^3,4^. The ability to discriminate between lineages within the PSSC and rapidly predict potential pathogenicity of novel lineages is crucial for preventing epidemic outbreaks^5^, detecting emerging pathogenic strains^6^, and untangling correlations between virulence factors carried by a pathogen, its host range, and its virulence^7^. Although the efforts to catalog PSSC diversity and to understand the molecular determinants of virulence have yielded great insights into their ecology and behavior^8^, currently there is no efficient way to leverage these insights to efficiently predict the identity and pathogenicity of newly discovered PSSC strains. This is especially true for those researchers or labs that do not specialize on PSSC.

**Fig. 1:**
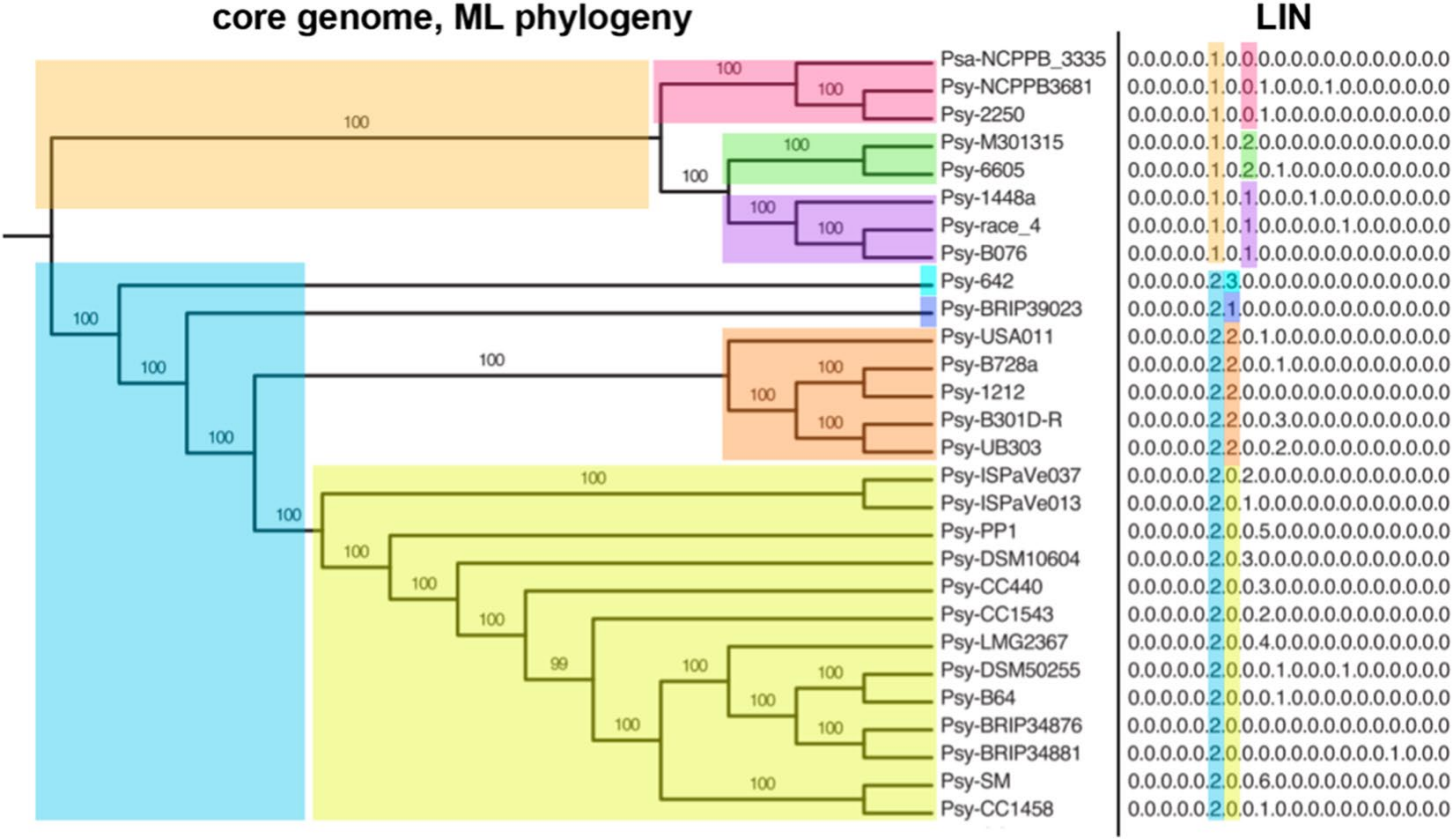
Comparison of clustering within PSSC that results from a maximum likelihood phylogenetic tree and LIN assigned based on ANI. Digits from left to right in each LIN correspond to inclusion of a strain in increasingly smaller clades within the phylogeny. Figure adapted from Vinatzer *et al*.^12^.

A major barrier to the characterization of PSSC strains is the inconclusive or inaccurate taxonomic identities of published genomes. By one estimate, 42% of all published PSSC genomes are misclassified at the species level, based on analysis of phylogenetic relationships described by average nucleotide identity (ANI) and multi-locus sequence analysis (MLSA)^9^. As genomes deposited in databases such as GenBank often serve as reference sequences for identification of isolates found on or near diseased plants, the high rate of misclassification has a direct, negative impact on our ability to efficiently recognize pathogenic lineages. Specifically, one of the most effective methods for classification of amplicon sequences is the naïve Bayes classifier^10^, which heavily relies on accurate training data to generate accurate predictions. The designation of 13 phylogroups based on MLST has clarified phylogenetic relationships within PSSC^11^, however most published genomes aren’t ascribed to a phylogroup in public databases and thus their use in classification is limited. While Berge *et al*. 2014^11^ have addressed this shortcoming by providing a reference database of phylogroup type strains allowing classification based on the CTS gene, there has since been no broader effort to make the classification process more efficient. Yet another approach to circumvent the inaccurate taxonomy at the species level while allowing for placement into clades below the species and phylogroup level is the clustering of genomes by ANI (Average Nucleotide Identity)^12^. This approach assigns a life identification number (LIN) to each unique genome in a database, creating hierarchical clusters of genomes that largely recapitulate traditional phylogenetic clades described by the core genome, and allow for higher resolution than traditional PSSC taxonomy (Fig. 1). Using LINs to generate an ANI-based taxonomy, we trained high resolution naïve Bayes classifiers for commonly used PCR primer sets targeting gyrB, gapA, CTS, rpoD^13^, and pgi^14^ (Table 1). As our classifiers report identity based on a difficult to interpret LIN, we also generated a comprehensive key describing key features for each of the 2,161 reference genomes in our training set along with their assigned LIN. This key allows for translation from classifier output to prediction of species, pathovar and phylogroups. As the vast majority of the genomes used in this study had no phylogroup assigned, we also provide new phylogroups assignments for over 2,000 publicly available PSSC genomes, based on previously suggested methods^11^.

**Table 1:** Primer sets accepted by Syringae.org for isolate characterization.

Target gene	Forward sequence (5′-3′)	Reverse Sequence (5′-3′)	primer names	Source
gapA	TCGARTGCACSGGBCTSTTCACC	GTGTGRTTGGCRTCGAARATCGA	gapA + 312 s/gapA−874 ps	Hwang *et al*.^13^
gyrB	TCBGCRGCVGARGTSATCATGAC	TTGTCYTTGGTCTGSGAGCTGAA	gyrB + 271 ps/gyrB−1022 ps	Hwang *et al*.^13^
CTS	CCTGRTCGCCAAGATGCCGAC	CGAAGATCACGGTGAACATGCTGG	gltA + 513 s/gltA−1130 s	Hwang *et al*.^13^
rpoD	GYGAAGGCGARATYGRAATCG	CCGATGTTGCCTTCCTGGATCAG	rpoD + 364 s/rpoD−1222 ps	Hwang *et al*.^13^
PGI	GCGTACTACCGYAMYCCBTC	CCACATMGGRAARATRTTYT	pgi	Yan *et al*.^14^

A second barrier to characterization of new PSSC isolates, even once identified, is the functional diversity exhibited throughout the species complex. Specifically, host range and virulence can vary considerably among pathogenic strains belonging to the same pathovar, while strains belonging to different pathovars can nonetheless exhibit similar host ranges. These complex patterns stem, at least in part, from the formal definition of pathovar as ‘a strain or set of strains with the same or similar characteristics, differentiated at infrasubspecific level from other strains of the same species or subspecies on the basis of distinctive pathogenicity to one or more plant hosts’^15^. This definition leaves room for broad interpretations of what should be considered a distinct pathovar. As such, some pathovars, such as pv. *avii*, have been delineated due to their ability to cause disease on a single host^16^, while pathovars are defined based on their different host ranges among a small defined group of hosts (*P. savastanoi* pvs. *savastanoi*, *nerii*, *fraxini*, *mandevillae* and *retacarpa*)^17^. Additionally, it has also been argued that pathogens sharing a wide common host range, regardless of a shared pathogenic potential for any single host, should also be considered as belonging to a single pathovar^18^. Given the inconsistent criteria for delineating between pathovars, and recent evidence that host ranges in PSSC strains overlap with no discernable modularity^19^, some groups have called into question the validity of pathovar designations for epidemiological and disease management purposes^20^. Further, properly assigning a given isolate to an appropriate pathovar requires performing host range tests that are prohibitively laborious to many labs.

An alternative phylogenomic approach to predicting pathogenic potential would be beneficial, as others have demonstrated that comparative genomics can discriminate between strains known to have different host ranges^21^ and correctly identify strains capable of infecting a given host^22^. In both of the above cases, presence of virulence factors, particularly those associated with the type III secretion system (T3SS), were highly correlated with known virulence patterns. Assuming T3SS effector proteins are conserved at some phylogenetic level, these results indicate that a phylogenomic signal may be present in PSSC that could be useful for assessing pathogenic potential without laborious experimental assays. In a recent contribution we showed the validity of such an approach by accurately predicting the presence of 77 type III effector (T3E) subfamilies in PSSC with a median accuracy of 80% using only single amplicon sequence data^23^. We provide here a dataset for ANI based interpretation of taxonomy of PSCC, a HMMER-based survey of known virulence factors associated with the T3SS, type 3 effectors (T3E), and the Woody Host and Pseudomonas (WHOP) region associated with woody host infection^24^ among our training set of genomes. With these data, we aim to provide a means for preliminary assessment and hypothesis generation regarding virulence traits from cost-effective amplicon sequencing data.

## Methods

### Reference PSSC genomes

**Fig. 2:**
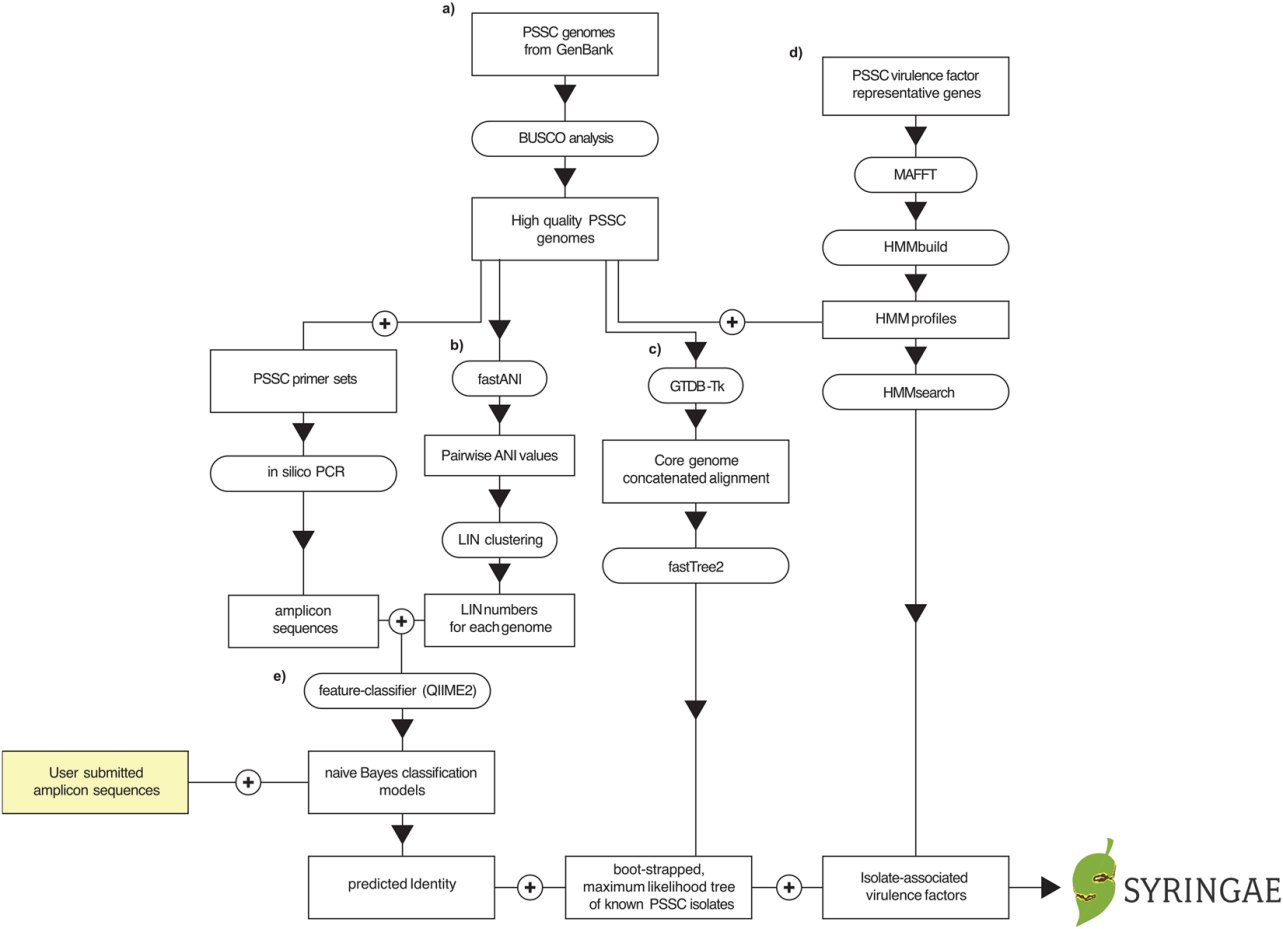
Schematic of bioinformatic pipeline used for generating dataset and classifiers, including their incorporation into a web portal for accessing dataset and classifiers – syringae.org.

All genome assemblies classified as ‘*Pseudomonas syringae* group’ (taxid 136849) were downloaded from the GenBank via NCBI in November 2021, resulting in 2,468 RefSeq records recovered^25^. Genomes were checked for completeness and assembly quality with BUSCO v5.3.1 using the pseudomonadales_odb10 lineage database^26^, and genomes with a BUSCO score ≥99 were kept for further processing (Fig. 2a). A CSV file (metadata.csv)^27^ summarizing each genome (and used as a backend database at www.syringae.org) was generated. Data included in this file are NCBI-submitted taxonomic data, type strain designations, phylogroups as assigned in this study, LIN clusters assigned for classification purposes, presence/absence of key virulence factors, and metadata found in each genome’s Biosample record.

### Assigning phylogroups to genomes

Phylogroup assignment of each genome was based on ANI shared with previously classified reference strains representing Phylogroups 1a,1b,2a,2b,2c,2d,3,4,5,6,7,9,10,11, and 13^28^ (Table 2). Reference strains for phylogroups 8 and 12 were not found among the 2,161 genomes characterized by SYRINGAE, either because they were not represented in the GenBank database or did not make it past the BUSCO quality check described above.

**Table 2:** Reference genomes used for phylogroup assignment.

RefSeq accession	Phylogroup
GCF_000172895.1	1a
GCF_001910465.1	1b
GCF_000145825.2	2a
GCF_003698965.1	2b
GCF_000177515.1	2c
GCF_003205905.1	2d
GCF_000012205.1	3
GCF_000156995.2	4
GCF_016599635.1	5
GCF_008692855.1	6
GCF_000452485.1	7
GCF_000452825.1	9
GCF_000452665.1	10a
GCF_000452785.1	10b
GCF_900104015.1	11
GCF_000452865.1	13

A genome was assigned to a given phylogroup if it was the most closely related to the reference strain for that phylogroup, based on ANI. To minimize inaccurate phylogroup assignments, 175 genomes sharing less than 95% ANI to any reference strain were left unassigned. These genomes might reflect understudied groups within PSSC, or genomes mischaracterized as PSSC. Further work beyond the scope of this study would be needed to properly account for their true identity.

### Assigning LIN clusters to genomes

A significant barrier to PSSC classification is unreliable and inconsistent taxonomic assignments. As such, SYRINGAE utilizes hierarchical clustering based on ANI values as an alternative to the Linnean taxonomy files typically used for Bayesian classification. Pairwise ANI between all genomes was calculated using fastANI v1.33 with default settings. Using the algorithm previously described^12^, each genome was assigned to LIN cluster (Fig. 2b). To describe the algorithm briefly, a random genome was designated as belonging to group ‘0’ at every ANI bin (e.g. assuming ANI bins of 80, 90, and 95% would give a LIN number of ‘0.0.0’). Each subsequent randomly selected genome was assigned a LIN number based on the genome it has the highest ANI with among genomes already assigned a LIN number. If, for example, the second genome selected had an ANI of 92% with the first genome, its LIN number would be assigned as ‘0.0.1’, as it meets the threshold for belonging to the same group as the first genome at the 80 and 90% ANI levels but differs from the first genome at the 95% level, and so a new group ‘1’ is created for it. All genomes were sequentially assigned LIN numbers in this way. For SYINGAE, ANI bins at 1% increments between 80–99% were used.

A drawback to using LIN clustering for classification is that the LIN number assigned to a given genome is highly dependent on the order of genomes selected for clustering (i.e. unless the same set of genomes is used and the order that these genomes are selected for clustering is preserved, the genomes are assigned different LIN numbers every time). Thus, classification models built with our LIN ‘taxonomy’ will always return LIN numbers that can only be interpreted when used in conjunction with a database that explicitly describes the genome each LIN number represents. We overcome this limitation by first providing such an interpretive database in the provided ‘metadata.csv’ file^27^ as well as by lowering the barrier to use with syringae.org, which uses metadata.csv to translate classification results automatically and the display classification results to the user using traditional taxonomic nomenclature and an interactive phylogenetic tree.

### Building the PSSC Phylogenetic tree

As a key component of visualizing and exploring the classifiers and dataset through the online portal hosted at www.syringae.org, a concatenated and masked gene alignment based on the core genome of PSSC was constructed using 120 bacterial marker genes within the BAC120 marker gene set with GTDB-TK 2.1.1 (using the ‘identify’ and ‘align’ commands)^29^. From this alignment, FastTree2^30^ with default settings was used to construct an approximately maximum-likelihood phylogenetic tree from nucleotide sequences (Fig. 2c).

### Screening genomes for virulence factors of concern

We generated a single HMM file containing HMMs for all virulence factors of concern (VFOC). This HMM file can be found in the data record VFOC.hmm. As a first step, representative gene sequences were gathered as follows:

Canonical T3SS: nucleotide sequences from PSSC strains DC3000 (GCF_000007805.1) and B728a (GCF_000012245.1), as annotated by NCBI (and available in data record ‘canonicalT3SS.fasta’) were used as a database along with the ‘annotate from database’ tool within the Geneious prime 2019 software package^31^, using 85% identity threshold for annotation of T3SS genes in all 2,161 genomes.

WHOP: previously annotated nucleotide sequences in strain NCPPB 3335^24^ were used as a database along with the ‘annotate from database’ tool within the Geneious prime 2019 software package^31^, using 85% identity threshold for annotation of WHOP genes in all 2,161 genomes.

T3E genes: T3E nucleotide sequences contained in PsyTEC^32^ were obtained from David Guttman on September 17^th^, 2021.

For each gene, nucleotide sequences from the above homologue search were aligned with MAFFT^33^ using default settings, and alignments were used as input for creation of HHM files using HMMER v3.3.2^34^ (Fig. 2d).

The VFOCs detailed in data records GENOME_VFOC.json and PROTEIN_VFOC.json are those that were found using the above HMM models. HMMER output files were manually inspected and filtered by E-value, with an E-value < 10^−20^ were considered to be statistically significant hits. In instances where two genes were identified as more than one virulence factor (a common occurrence among closely related T3E subfamilies), the identification with the lowest E-value was chosen as the official annotation.

### PSSC primer set selection

Over the last two decades, several PCR primers have been developed, often as part of MLST schemes, for building evolutionary accurate phylogenies and aiding in classification of unknown isolates. More recently, there has been interest in utilizing single amplicon sequences for these purposes. To investigate which primer sets provide the most value for classification using a single amplicon, we conducted a short but thorough in-silico investigation of 16 commonly used primer sets^23^. Briefly, we assessed in-silico amplification in 2,161 genomes representing the full diversity of the species complex as we currently know it, investigated concordance between pairwise amplicon distance and whole genome ANI, and assessed resolution of naïve Bayes classifiers trained from amplicon data, as well as the potential for functional prediction based on the classification results. The best performing primer sets based on these metrics are represented in the classifiers presented here (see Table 1).

### Training Naïve Bayes classification models

For each marker gene, a classification model was trained using the scikit-learn v0.24.1 feature-classifier plugin in QIIME 2 v2020.8.0. Training naïve Bayes classifiers requires both a list of sequences, and an associated taxonomy file for each sequence (typically in the format ‘Order_Pseudomonadales; Family_ Pseudomonadaceae; Genus_Pseudomonas; Species_syringae;’). LIN numbers assigned to each genome were used to construct a hierarchical taxonomy, with ANI bins within each LIN number acting as taxa levels, and groups acting as individual taxa (e.g., a taxonomy format of ‘80%_0; 90%_0; 95%_1) (Fig. 2e). in silico amplicon sequences and the LIN taxonomy file used for training classifiers can be found in data records ‘LIN_taxonomy.tsv’ and the five FASTA files labeled in accordance with the primer sets outlined in Table 1.

## Data Records

All necessary data are deposited at Zenodo^27^.

Data include:

*In silico amplicon sequences*

FASTA files containing sequences, used as input for training classifiers.

(CTS_Hwang.fasta, gapA_Hwang.fasta, gyrB_Hwang.fasta, pgi_Yan.fasta, and rpoD_Hwang.fasta)

*LIN_taxonomy.tsv*

LIN numbers associated with each reference genome, used as input for training classifiers

*Classifiers*

Qiime 2 classifier artifacts for each PCR primer set listed in Table 1. Classifiers take as input untrimmed amplicon sequences and return predicting LIN groups.

*Metadata.csv*

The metadata file links LIN groups assigned to each reference genome used to train classifiers with their species, phylogroup, pathovar, and virulence factors. Columns are described in Table 3

**Table 3:** Description of the data file metadata.csv.

Column name	Description
name	RefSeq genome accession
Type strain	Status as type or pathotype strain
Submission Date	Date of NCBI genome submission
Submitting Organization	Submitter’s identified organization
Geographic Location	Submitter’s identified Geographic location for organism
Isolation Source	Submitter’s identified source of isolation
Organism	Submitter’s identified full organism identity
Species	Submitter’s identified species
Pathovar	Submitter’s identified pathovar
Strain	Submitter’s identified strain
Assembly name (Alt. strain)	Submitter’s identified assembly name – used for strain name when strain not given
Taxonomy check	Result of NCBI’s taxonomy check based on Submitter’s identification
antA – HopBJ1	Number of genes identified by HMMER as the indicated virulence factor. Specific protein accessions and e-values for each and can be found by cross-referencing column name and/or genome accession within VFOC.json files
ANI_80 – ANI_99	LINs at each ANI level. To be used for interpreting classification output
Phylogroup	as assigned by this work

*VFOC.hmm*

*Contains HMM files useful for screening whole genomes for canonical T3SS, T3E family, and WHOP genes*.

*PROTEIN_VFOC.json and GENOME_VFOC.json*

The VFOC files describe all canonical T3SS, T3E, and WHOP genes in the 2,161 genomes used in this dataset, as detected by HMMER, in an easy to query JSON format. Each file contains the same data, with RefSeq protein (*PROTEIN_VFOC.json)* and genome accessions (*GENOME_VFOC.json)* as top-level keys. Additional keys found in each file are described in Table 4.

**Table 4:** Description of the data files GENOME_VFOC.json and PROTEIN_VFOC.json.

PROTEIN_VFOC.json	
Key	Value
genomes	List of RefSeq accessions that contain the protein accession described by the top-level key
HMMER	Virulence factors ascribed to this protein accession by HMMER
annotation	NCBI annotation of this protein accession
**GENOME_VFOC.json**	
**Key**	**Value**
accession	RefSeq protein accession
E-value	Virulence factor identification confidence, as reported by HMMER
annotation	NCBI annotation of this protein accession

*PSSC.tree.txt*

A newick tree file describing a core genome phylogeny of genomes used in this work.

## Technical Validation

Genome records used in creation of this dataset were validated for assembly quality using BUSCO (ref), and all genomes with a reported BUSO score <99 were removed from the dataset. Accuracy of the classification models and functional predictions were investigated and published separately^23^. Beyond the T3SS, T3E, and WHOP genes, which were annotated using HMM models built for this study, all gene annotations were taken directly from the NCBI Prokaryotic Genome Annotation Pipeline.

## Usage Notes

The data described in this work are also available within a functional tool - syringae.org (usage described in Supplementary Figures 1-4, Supplementary File 1)

### Supplementary information

Supplementary File 1

## Data Availability

All scripts used in the generation of classifiers and dataset, as well as source code for the web app hosted at syringae.org are available on GitHub at https://github.com/cwf30/SYRINGAE^35^. To aid in reproducibility, a conda environment YAML file (SYRINGAE_env.yml) and a readme file outlining scripts used (README.txt) are also provided.
