# Supplementary material for: Naïve Bayes Classifiers and accompanying dataset for Pseudomonas syringae isolate characterization
Source: Sci Data. 2024 Feb 7;11:178. doi: 10.1038/s41597-024-03003-x (PMC10850129; doi:10.1038/s41597-024-03003-x)
Supplement: Supplementary file 1 [file 41597_2024_3003_MOESM1_ESM.pdf]

Supplementary File 1

Table of contents

Usage Notes for Syringae.org …. 2
Supplemental figure 1 ……………….. 3
Supplemental figure 2……….……….. 4
Supplemental figure 3………………… 5
Supplemental figure 4 ……………….. 6

Usage Notes For syringae.org

There are three main functions of the web applications, Identification, phylogenetic exploration, and PSSSC genome/gene search. Each of these functions is described below.

1) Identify

The primary functionality on syringae.org is the rapid characterization of *Pseudomonas sp.* isolates from single amplicon sequences.

Input:

The query sequence(s) are untrimmed amplicon sequences generated from primer sets targeting the genes gyrB, CTS, gapA, PGI, or rpoD, in FASTA or multiFASTA format.

Outputs:

1) A list of genomes predicted to be most closely related to the unknown isolate

2) Phylogenetic classification for each query sequence is also displayed as a phylogenetic tree rooted at the most recent common ancestor of all genomes in the Syringae database predicted to be closely related to the unknown isolate. By default, the tree only shows the most closely related genomes, but users can toggle the ANI threshold scale to lower values, to “zoom out” and display more distant relatives. This extra functionality allows the ability to visualize the predicted placement of the unknown isolate in a larger phylogenetic context. The predicted shared ANI with the unknown isolate is displayed as a radial bar chart around the perimeter of the tree (Supplemental fig. 1, external ring).

3) For the currently displayed tree, the abundance of represented species, phylogroups, and pathovars are to the left of the tree. (Supplemental fig. 1)

4) A summary of the virulence factors found among the most closely related of the unknown isolate genomes given the selected ANI threshold is available as a second tab in the results section. (Supplemental fig. 2)

To quickly learn more about any genomes predicted to be similar to the unknown isolate, users can utilize the Explore and Search functionalities.

2) Explore

An interactive visualization tool for exploring the phylogenetic and genetic diversity of PSSC. Users can filter the 2,161-genome phylogenetic tree Syringae uses for visualizing classification data by taxa

(phylogroup, species, and pathovar), and annotate up to six features, including multiple taxa and presence/absence of up to 3 NCBI-annotated genes (Supplemental fig. 3)

3) Search

A search tool for quickly finding metadata for any genome in SYRINGAE's database. Search results include a list of the closest PSSC relatives in the dataset, as measured by fastANI (Supplemental fig. 4).

Users can also search for any NCBI-annotated gene names or virulence factors annotated by us for Syringae. Search results include a list of all genomes in our database carrying the gene of interest, as well as each unique protein accession number associated with the gene name found within the species complex.

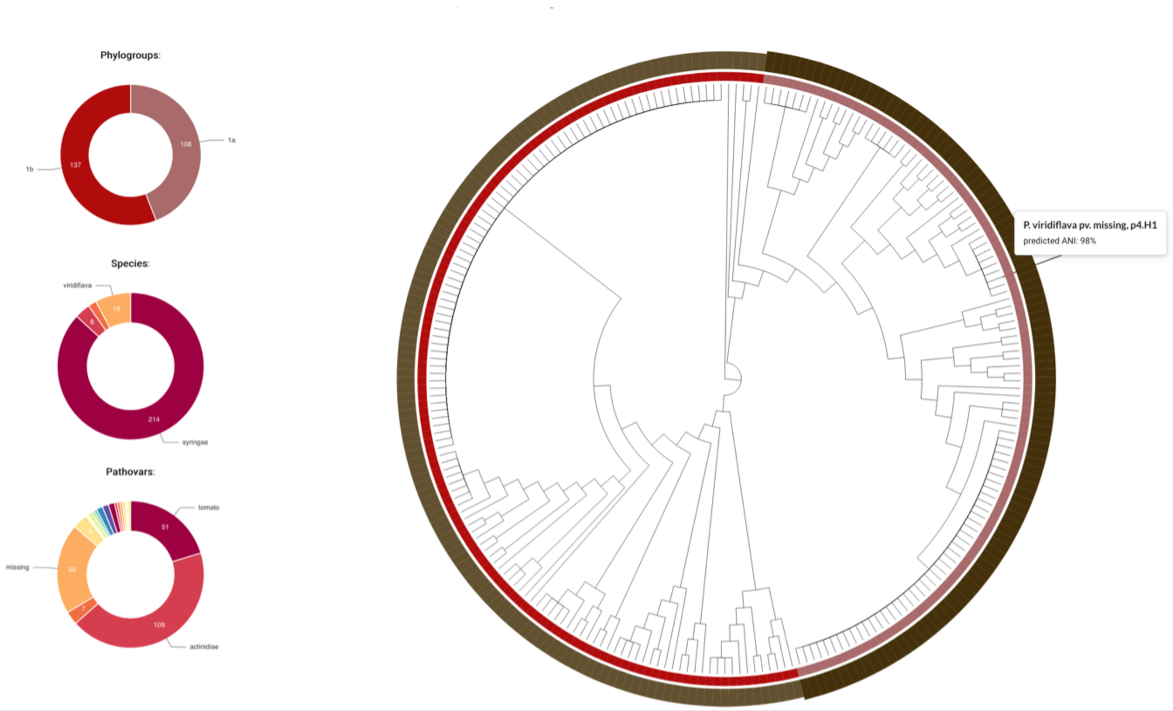

Supplemental figure 1 A screenshot of classification results provided by Syringae.org. The tree is rooted at the most recent common ancestor of all genomes within the LIN cluster the unknown isolate has been placed into. The exterior ring shows the predicted ANI similarity between the unknown isolate and each reference genome. The interior ring shows phylogroup assigned to each reference genome. Relative abundance of phylogroups, species, and pathovars found within the unknown isolates predicted LIN cluster are shown to the left of the tree.

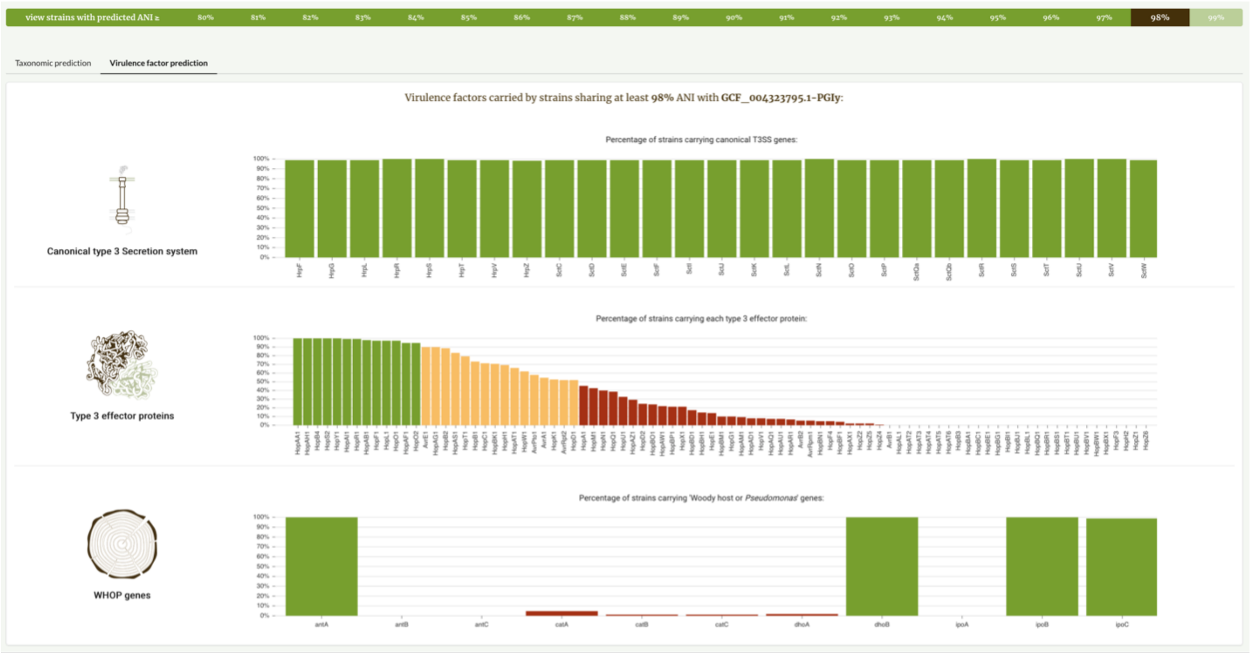

Supplemental figure 2 A screenshot of virulence factor prediction provided by Syringae.org. The proportion of genomes within an unknown isolate's predicted LIN cluster that carry canonical type III secretion system genes, type III effectors, and WHOP genes are displayed. Green, yellow, and red bars denote virulence factors found in >90%, >50%, and <50% of related genomes, respectively.

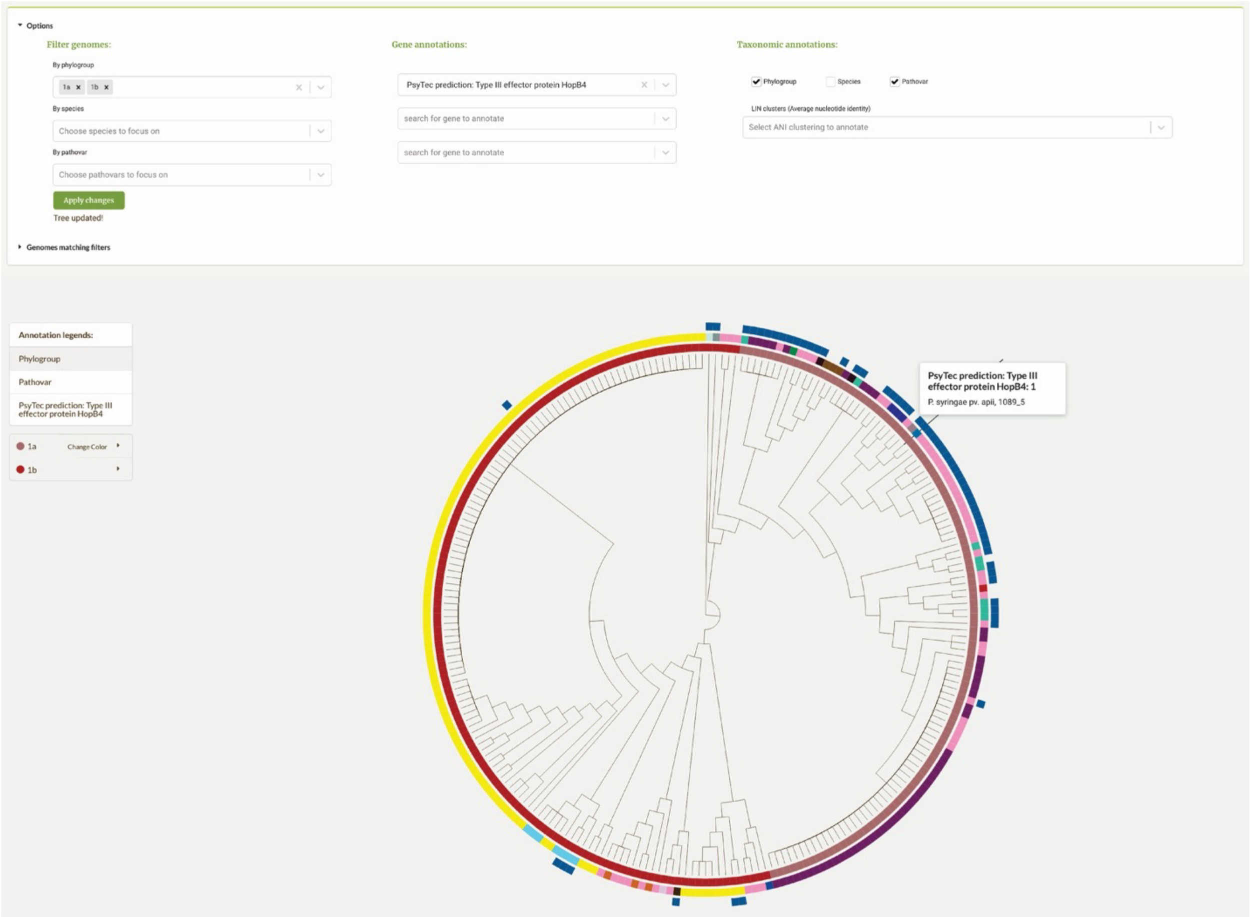

Supplemental figure 3 A screenshot of the Explore functionality on Syringae.org. The form at top allows for filtering of genomes to display by taxa, and annotation by up to 3 simultaneous genes and 4 taxa. Annotation rings surrounding the phylogenetic tree are based on user-selected annotations; in this case showing, from innermost ring outward, phylogroups, pathovars, and absence/presence of effector protein subfamily HopB4.

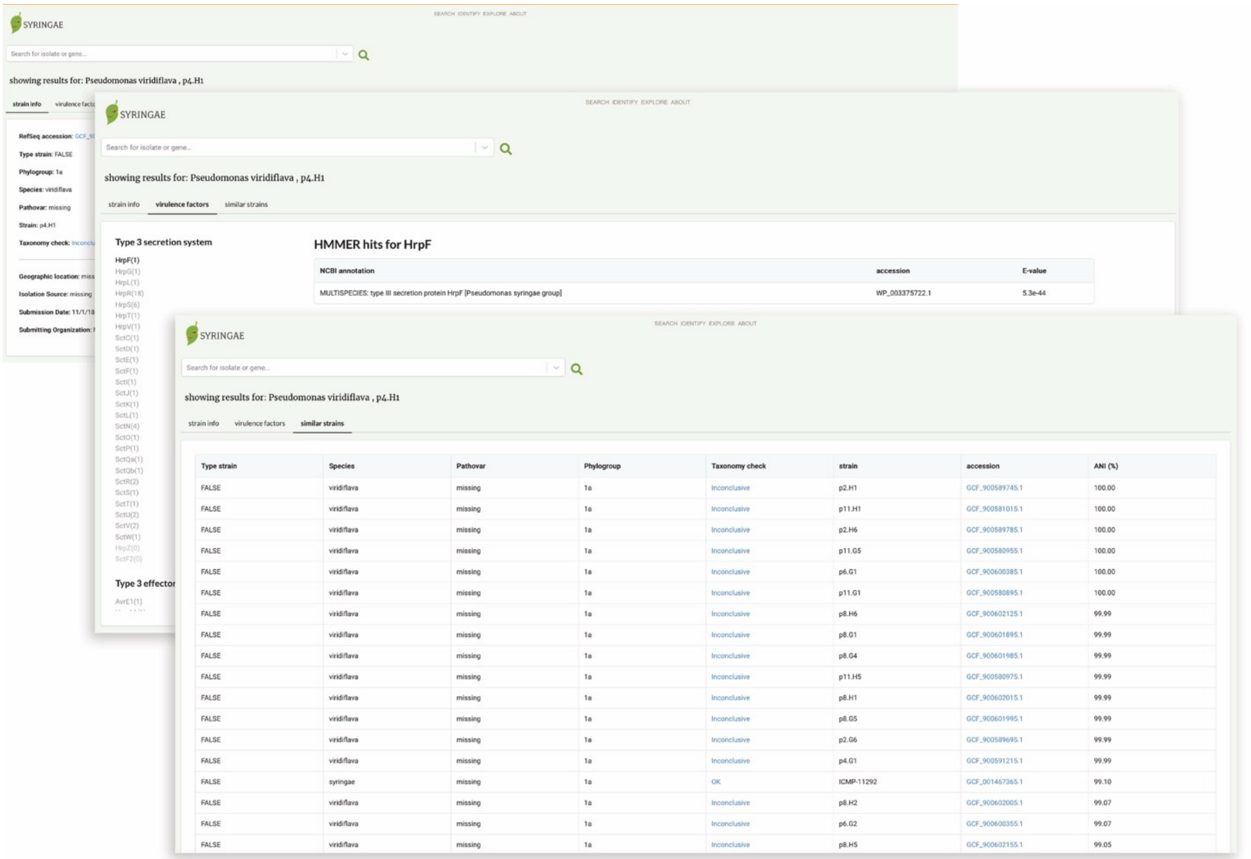

Supplemental figure 4 A screenshot of search results for a PSSC genome displaying from top to bottom: metadata associated with the genome, predicted virulence factors, and most closely related genomes.
